# Quantification of *Clostridioides (Clostridium) difficile* in feces of calves of different age and determination of predominant *Clostridioides difficile* ribotype 033 relatedness and transmission between family dairy farms using multilocus variable-number tandem-repeat analysis

**DOI:** 10.1186/s12917-018-1616-8

**Published:** 2018-10-01

**Authors:** Petra Bandelj, Céline Harmanus, Rok Blagus, Marko Cotman, Ed J. Kuijper, Matjaz Ocepek, Modest Vengust

**Affiliations:** 1 Veterinary faculty, University of Ljubljana, Cesta v Mestni log 47, SI-1115 Ljubljana, Slovenia; 2 Department of Medical Microbiology, Center of Infectious Diseases, Leiden University Medical Center, Leiden, Netherlands; 3 Institute for biostatistics and Medical informatics, University of Ljubljana, Vrazov trg 2, SI-1104 Ljubljana, Slovenia

**Keywords:** *Clostridioides (Clostridium) difficile*, Ribotype 033, community-acquired infection, Dairy cattle, Epidemiology

## Abstract

**Background:**

Community acquired *Clostridioides (Clostridium) difficile* infection (CA-CDI) is a significant health problem in human and veterinary medicine. Animals are often considered as potential reservoirs for CA-CDI. In Europe, family farming is the most predominant farming operation, with a complex interaction between animals and the community. Therefore, it is pertinent to evaluate transmission patterns of *C. difficile* on such prominent European farming model.

Fecal samples from calves (*n* = 2442) were collected biweekly over a period of one year on 20 mid-size family dairy farms. Environmental samples (*n* = 475) were collected in a three month interval. *Clostridioides difficile* was detected using qPCR in 243 fecal samples (243/2442); positive samples were then quantified. Association between prevalence/load of *C. difficile* and age of the calves was estimated with logistic regression model. Most common *C. difficile* isolate from calves (*n* = 76) and the environment (*n* = 14) was *C. difficile* ribotype 033, which was further analyzed using multilocus variable-number tandem-repeat analysis (MLVA) to assess intra- and between-farm relatedness.

**Results:**

*Clostridioides difficile* was detected in feces of calves less than 24 h old. Results showed a non-linear statistically significant decrease in shedding load of *C. difficile* with age (*P* < 0.0001). A nonlinear relationship was also established between the number of calves and the farm *C. difficile* prevalence, whereas the prevalence of *C. difficile* ribotype 033 increased linearly with the number of calves. MLVA revealed close intra-farm relatedness among *C. difficile* ribotypes 033. It also revealed that the between-farms close relatedness of *C. difficile* ribotypes 033 can be a direct result of farm to farm trade of calves.

**Conclusions:**

Implementation of better hygiene and management measures on farms may help decrease the risk of spreading CA-CDI between animals and the community. Trading calves older than 3 weeks would decrease the possibility *C. difficile* dissemination in the community because of lower prevalence and lower load of *C. difficile* in feces.

**Electronic supplementary material:**

The online version of this article (10.1186/s12917-018-1616-8) contains supplementary material, which is available to authorized users.

## Background

The increasing number of community acquired *Clostridioides difficile* infection (CA-CDI) in the past decade has prompted investigations into animal source of CDI [1, 2]. Several studies have shown that *Clostridioides difficile* can cause gastrointestinal disease in dogs, horses, piglets and possibly calves [3–9]. It remains unclear, however, if *C. difficile* strains found in animals can cause CDI in humans [9].

Family dairy farms were included in this study. Family farms are the most common operating farming system in the European Union. It represents the sustainable agriculture/agribusiness, and is beneficial for the local community [10]. To estimate the significance of a possible infection source, shedding numbers/load of *C. difficile* had to be quantified and *C. difficile* strains phylogenetically assessed. One of the major risk factor for increased *C. difficile* shedding in the environment is the age of the animal [11–14]. Regardless of the farming management, the prevalence of *C. difficile* has been shown to decrease dramatically with age from 10 to 56% at and near birth, to 0–3.8% at the time of slaughter [11, 14–16].

Several studies evaluated the impact of *C. difficile* shedding with calves feces by reporting *C. difficile* prevalence [8, 17–20], using longitudinal models [11, 14–16], or more recently, enumeration with viable plate counts from feces and carcasses of newborn calves [21]. None of them, however, quantified *C. difficile* in calves in relation to their age, which would indicate the age related risk for *C. difficile* dissemination in the community.

Calves on family farms have been shown to harbor several *C. difficile* ribotypes, with *C. difficile* ribotype 033 being the most prevalent [22]. Most previous reports suggest that *C. difficile* ribotype 033 is of less clinical importance compared to ribotypes 078, 027, 014 and 012, which are frequently isolated from feces of calves raised in big veal raising operations [6, 11, 14, 15, 18, 23, 24]. In humans, due to different genotype and phenotype, ribotype 033induced CDIs might be underdiagnosed [25]. However, one report has associated *C. difficile* ribotype 033 with diarrhea and eventually death in an elderly hospitalized patient in Italy [26], which necessitates further epidemiological assessment of all *C. difficile* ribotypes. Detailed epidemiological investigation can be achieved employing multilocus variable-number tandem-repeat analysis (MLVA), which is the method of choice to identify routes of transmission between patients and the environment [27–29]. This method shows a high level of discrimination and was proven useful for geographical tracking of several outbreak strains of bacteria [30–32].

The aim of this study was to quantify *C. difficile* in calves’ feces from birth to six months of age, to determine how much calves contribute to the shedding of *C. difficile* into environment, and to evaluate the relatedness of the most predominant *C. difficile* ribotype 033 between family dairy farms.

## Results

**Fig. 1:**
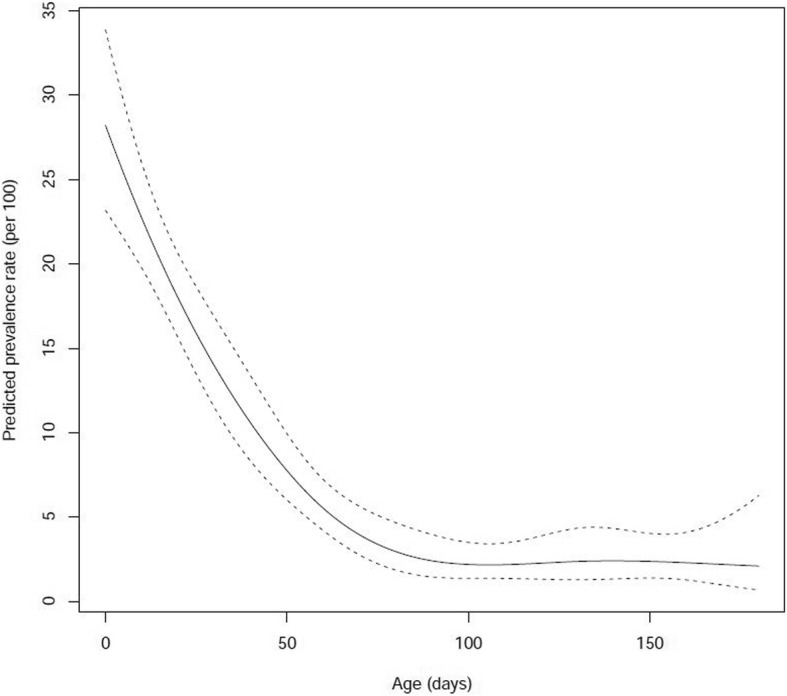
*C. difficile* prevalence rates in calves from 0 to 180 days old per 100 and 95% confidence intervals

Overall, 243 fecal samples positive for *C. difficile* were collected from 155 calves. A hundred and seven calves (*n* = 107/155, 69%) were positive only once (Additional file 1 and Additional file 2). Forty eight calves (*n* = 48/155, 31%) were positive multiple times (2-6×) (Table 1). *Clostridioides difficile* prevalence in calves feces decreased significantly with the increasing age of the calves (*P* < 0.0001) (Fig. 1).

**Table 1:** Quantification results for *C. difficile* in feces of calves that were positive multiple times

Calf										
1	Age (days)	10	24	38	52	66	80	94	108	
	No. CD	102	0	0	0	0	0	88	0	
2	Age (days)	11	25	39	53	67	81	95		
	No. CD	0	3716	0	0	0	LOQ	0		
3	Age (days)	12	27	40	54	68	82			
	No. CD	147	578	0	LOQ	194	0			
4	Age (days)	10	24	38	52	66				
	No. CD	0	0	318	174	0				
5	Age (days)	6	20	34						
	No. CD	2758	732	0						
6	Age (days)	5	19	33	47					
	No. CD	0	3300	110	0					
7	Age (days)	2	16	30	44					
	No. CD	684	0	LOQ	0					
8	Age (days)	3	17	31	45	59				
	No. CD	295	0	0	LOQ	0				
9	Age (days)	2	16	30						
	No. CD	1530	441	0						
10	Age (days)	5	19	33	47	61	75			
	No. CD	135,486	92	0	183	LOQ	0			
11	Age (days)	5	19	33	47	61	75	89	103	117
	No. CD	2676	572	241	0	209	0	0	LOQ	0
12	Age (days)	9	23	37	51	65				
	No. CD	805	0	942	129	0				
13	Age (days)	9	22	36	50	92	106	120		
	No. CD	662	408	1189	0	0	610	0		
14	Age (days)	1	15	29	43	57	71			
	No. CD	96,398	37,640	0	0	LOQ	0			
15	Age (days)	11	25	39	53	67				
	No. CD	3569	2683	1901	LOQ	0				
16	Age (days)	1	15	29	43	57				
	No. CD	13,705,988	32,219	1623	368	0				
17	Age (days)	0	14	28	42	56	70	84		
	No. CD	1519	0	LOQ	0	41	198	0		
18	Age (days)	0	14	29	42	56	70	0		
	No. CD	0	0	333	0	274	0	0		
19	Age (days)	12	26	40	54	67	82			
	No. CD	0	0	890	65,507	2867	0			
20	Age (days)	59	73	87	101	115	129	143	157	171
	No. CD	0	242	0	0	0	0	0	LOQ	0
21	Age (days)	3	17	31						
	No. CD	3937	4115	0						
22	Age (days)	1	15	29	43	57	71	85		
	No. CD	114	0	287	0	0	356	0		
23	Age (days)	13	27	41						
	No. CD	23,470	322	0						
24	Age (days)	3	17	31						
	No. CD	54,900	4,278,134	0						
25	Age (days)	1	15	29	43	57	71	85	99	113
	No. CD	103,235	1177	0	0	0	0	0	LOQ	0
26	Age (days)	11	25	39	53	67	81	95	109	123
	No. CD	274,108	0	0	0	0	0	0	631	0
27	Age (days)	12	25	40						
	No. CD	10,811,054	938	0						
28	Age (days)	2	16	30	44	58	72			
	No. CD	0	105,727	0	0	LOQ	0			
29	Age (days)	9	23	37	51					
	No. CD	2919	0	LOQ	0					
30	Age (days)	7	21	35						
	No. CD	2,589,994	1152	0						
31	Age (days)	4	18	32	46					
	No. CD	0	806	38,702	LOQ					
32	Age (days)	7	21	35						
	No. CD	9630	1827	0						
33	Age (days)	6	20	34						
	No. CD	1,587,504	5278	0						
34	Age (days)	9	23	37	51	65	79	93		
	No. CD	3,906,984	1316	1055	0	0	783	0		
35	Age (days)	13	27	42	55	69	83	97		
	No. CD	53,735	66,424	LOQ	20,396	0	LOQ	0		
36	Age (days)	12	26	40	54	68				
	No. CD	842,730	1084	0	304	0				
37	Age (days)	3	17	31	45	59				
	No. CD	7,260,792	1346	632	LOQ	0				
38	Age (days)	5	19	33	47	61	75			
	No. CD	11,629	137	3715	0	723	0			
39	Age (days)	34	48	62	76	90				
	No. CD	294	0	0	LOQ	0				
40	Age (days)	9	23	37	51					
	No. CD	207	LOQ	LOQ	0					
41	Age (days)	10	24	38						
	No. CD	428	401	0						
42	Age (days)	6	20	34						
	No. CD	28,022	779	0						
43	Age (days)	20	34	48	62	76	90	104	118	132
	No. CD	0	30,971	0	0	0	0	0	0	LOQ
44	Age (days)	9	22	37						
	No. CD	3,593,368	4064	0						
45	Age (days)	8	21	36						
	No. CD	678,040	1627	0						
46	Age (days)	11	25	39	53	67	81			
	No. CD	129	13,724	0	0	537	0			
47	Age (days)	0	14	28	42	56				
	No. CD	0	3,200,151	7897	2160	0				
48	Age (days)	12	26	40						
	No. CD	48,901	8297	0						

*LOQ* under the limit of quantification; *CD Clostridioides difficile*

### Association between *C. difficile* prevalence and the number of calves

**Fig. 2:**
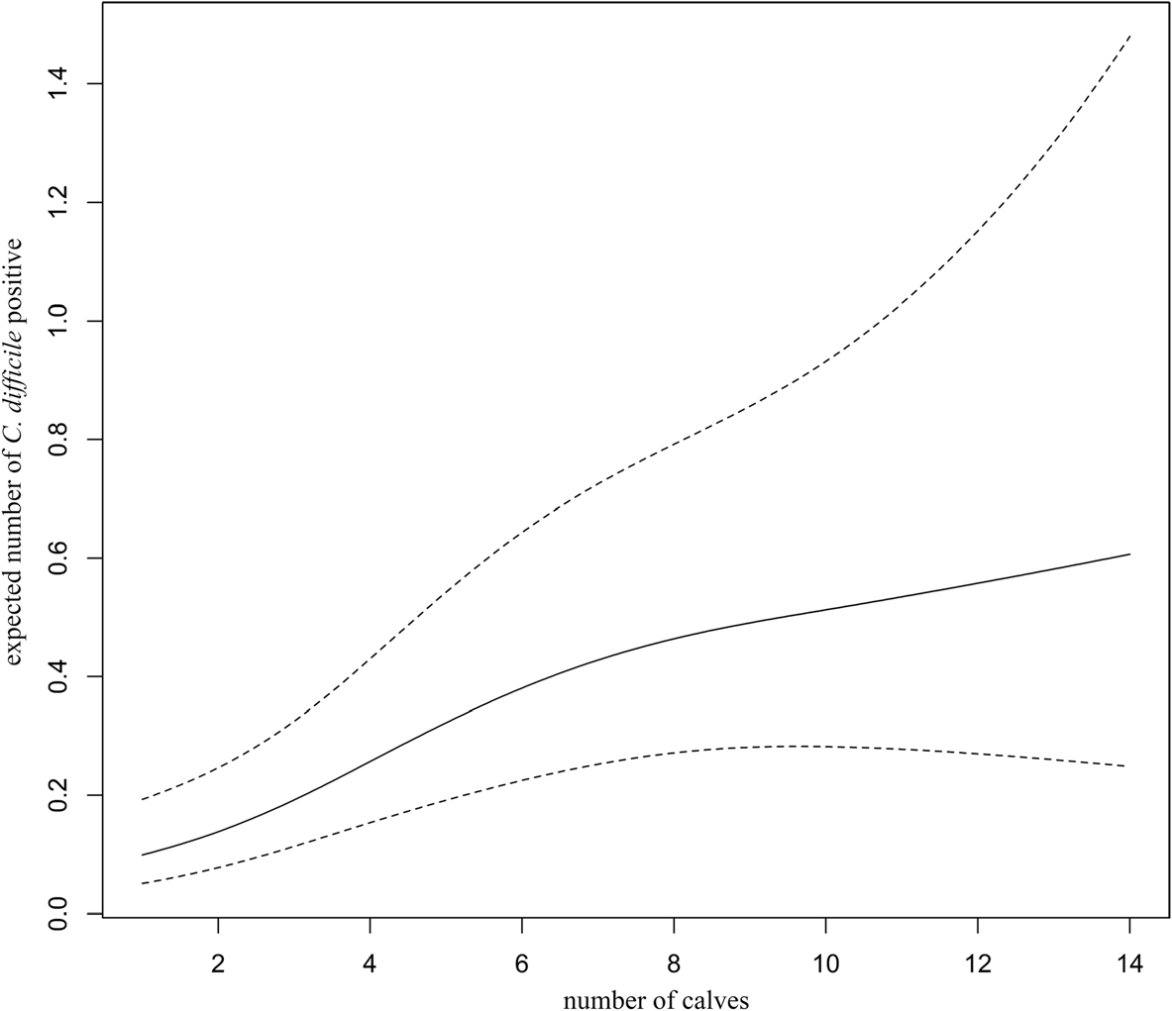
Expected prevalence of *C. difficile* positive calves versus the total numer of calves on the farms

A nonlinear association between the number of calves and the farm prevalence was established for *C. difficile* (*P* < 0.0001, Table 2). A steady increase in *C. difficile* prevalence is observed when the number of calves on the farm is up to eight (8), whereas for larger number of animals the *C. difficile* prevalence seems to be only mildly affected by the number of calves (Fig. 2). The prevalence of ribotype 033, however, increases linearly with the number of calves on a farm (*P* = 0.032, Table 2).

**Table 2:** The association between the prevalence of *C. difficile* and ribotype 033 and the number of calves

Outcome	Variable	Estimate	SE	*p*	(Exp)estimate	95% CI
*C. difficile*	Number of Calves			< 0.0001		
	linear	0.33	0.08	< 0.0001	1.39	1.18–1.66
	non-linear	−0.39	0.16	0.0174	0.68	0.48–0.93
	Time of sampling	0.02	0.01	0.0251	1.02	1.01–1.04
Ribotype 033	Number of Calves	0.13	0.06	0.0323	1.13	1.01–1.27
	Time of sampling	0.01	0.01	0.4932	1.01	0.98–1.04

The time of sampling is included only to control for the effect of the meteorolical season. The linear/non-linear effect of *C. difficile* prevalence should be estimated from Fig. 2

*SE* Standard error

### Quantification

**Fig. 3:**
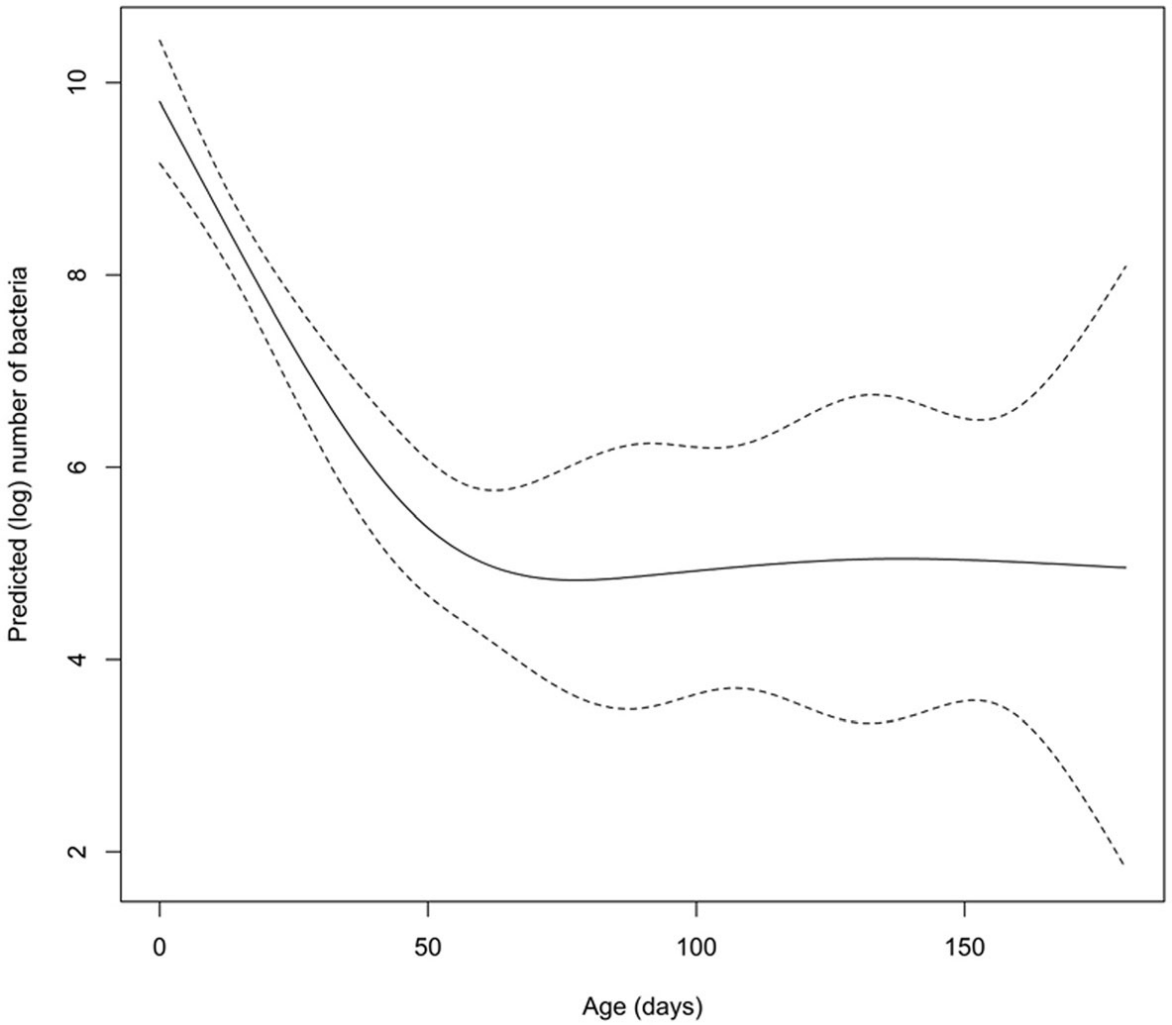
Predicted log transformed load of *C. difficile* and age (in days) of calves and 95% confidence intervals

The load of *C. difficile*/g of calves feces decreased with age (*P* < 0.0001) (Fig. 3). One day old calves had the highest load of *C. difficile* in feces (mean 3.4 × 10^6^ cells/g feces; 453–13.7 × 10^6^ *C. difficile* cells/g feces), followed by calves that were 8, 11 and 12 days old with the mean of 1.8–1.9 × 10^6^ cells/g feces (50–10.8 × 10^6^ *C. difficile* cells/g feces). Some calves (less than 24 h old) were also positive for *C. difficile* and had a mean of 6.2 × 10^3^ *C. difficile* cells/g feces (126–2.2 × 10^4^ *C. difficile* cells/g feces).

### MLVA

**Fig. 4:**
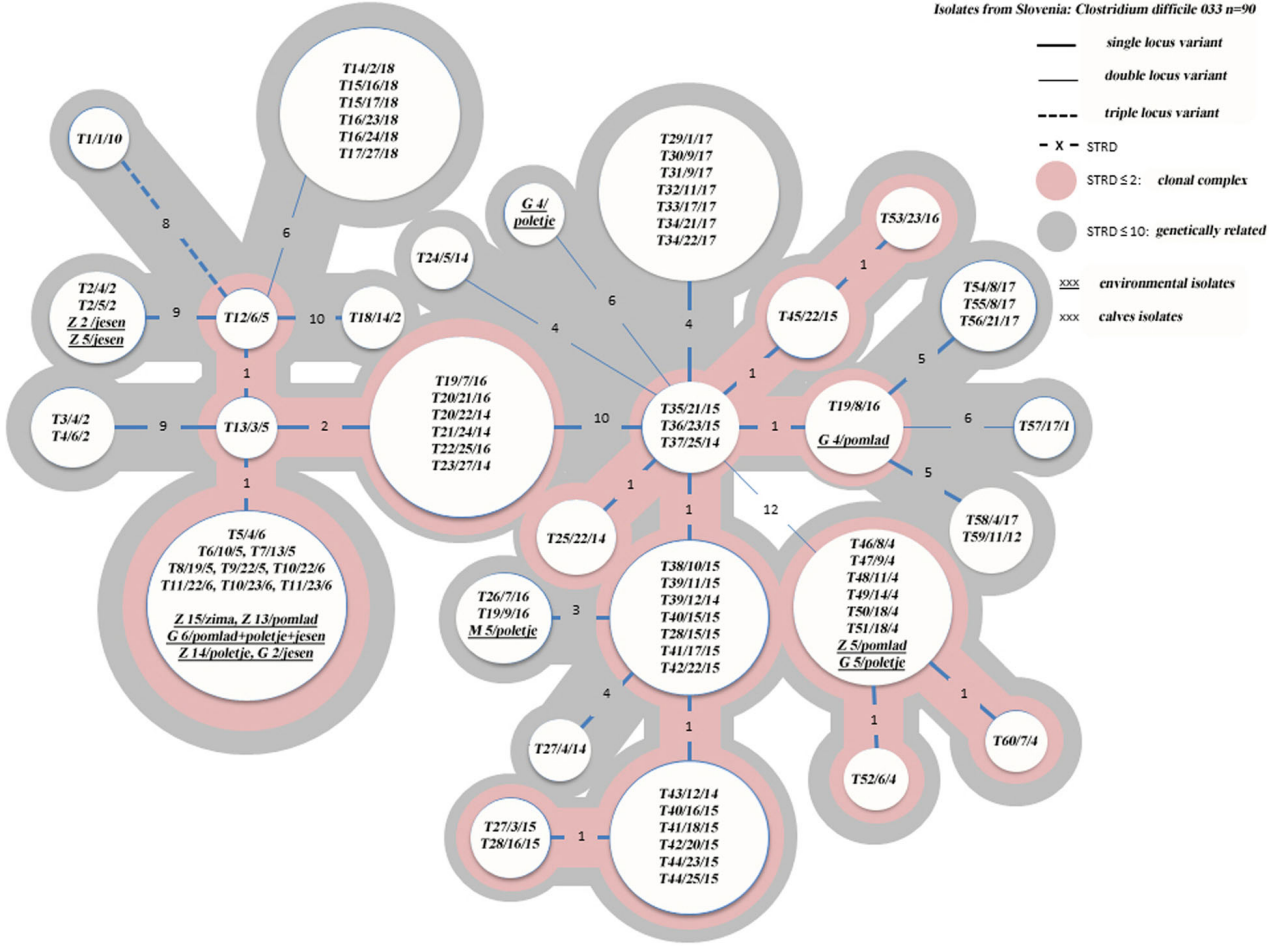
Minimum spaning tree for *C. difficile* ribotype 033 isolated from calves feces and environmental samples: Clonal (STRD = 0), clonal complex (STRD ≤2), genetically related (STRD ≤10). Legend: Tn/n/n – individual calf number/sampling time/farm; Z n – soil sample on farm n; G n – manure sample from farm n; M n – flies from farm n

The minimum spanning tree (Fig. 4) revealed close relatedness among most *C. difficile* ribotype 033. Isolates from calves from the same farm (farm 4, 17 and 18) or local community (farms 5 and 6; farms 14, 15 and 16) were mostly clonal (STRD = 0) or in one clonal complex (STRD≤2). Most calves that were positive for *C. difficile* ribotype 033 several times during the sampling period, harbored the same *C. difficile* clone (T2, T10, T11, T15, T16, T20, T34, T39, T44), had *C. difficile* isolates in the same clonal complex (T40, T41, T42), or were genetically related with STRD≤10 (T27, T28). Two male calves, T20 and T39, from farms 15 and 16 were sold to farm 14. Both were positive with the same clone of *C. difficile* ribotype 033 before and after their relocation. The *C. difficile* clone from calf (T20) was found subsequently in calves born on farm 14 (T21 and T23). Same clone was not present on farm 14 before introduction of calf T20. *Clostridioides difficile* ribotype 033 clone recovered from calf T39 was introduced from farm 15 to farm 14. However, the same clone wasn’t found in any other calves born on farm 14.

The same MLVA profile (*n* = 6) that was identified from the environmental samples, could be linked to the *C. difficile* ribotype 033 isolated form calves of the same farm or from the same geographical area (Fig. 4). Several (*n* = 7) were more genetically related to isolates from other unrelated farms. Interestingly, *C. difficile* ribotype 033 recovered from a barn fly on farm 5, had the same MLVA profile as the *C. difficile* recovered from calves (T19, T26) on an epidemiologically unrelated farm (farm 16).

## Discussion

The aim of the study was to quantify *C. difficile* in feces of calves and to evaluate the relatedness of the most common *C. difficile* ribotype 033 between economically related and unrelated family dairy farms.

We also established that the *C. difficile* prevalence is non-linearly related to the number of calves on the farm. While a steady increase in *C. difficile* prevalence is observed up to a certain number of animals, it then remains roughly at the same level, when the number of animals increases further. The prevalence of *C. difficile* ribotype 033, however, is linear to the number of calves on the farm.

Newborn calves had the highest prevalence and load of *C. difficile* /g feces, which decreased over time. The decrease in prevalence over the increasing age of animals has previously been established [11, 14]. However, this is the first report associating the fecal load of *C. difficile* with age of calves. Repeated sampling of the same calves over their first 6 months of life has also shown that calves can be positive for *C. difficile* more than once with the same or different ribotype or MLVA profile.

In this study calves aged 1 day and around 12 days had the highest load of *C. difficile* cells/g feces. Interestingly, some calves sampled in the first 24 h after birth, were also shedding *C. difficile* with feces (meconium). This is in concordance with Hopman et al. [33], who demonstrated CD shedding in piglets after their first hour of life and the prevalence at day one increased from 8.3 to 62%.

The predominance of *C. difficile* ribotype 033 on sampled farms gave us the opportunity to investigate the epidemiology of *C. difficile* between economically related and unrelated family dairy farms. Most studies to date were performed on big veal farms which congregate large numbers of calves of different geographical origins [11, 34]. Our study included only family farms located in a driving distance between each other (≤ 2 h from farm to farm), where some are economically connected with trading of calves or products. As expected the *C. difficile* isolates 033 from the same farm sampled on different dates or different calves were clonal or in the same clonal complex as was recently found also in piglets [35]. The similarity was also shown in *C. difficile* isolates from farm sharing the same private farm road (farm 5 and 6) or trading of the male calves (farm 14 and 15; farm 14 and 16). We even suggested a possible transmission of *C. difficile* from one farm (farm 16) to another (farm 14) through a colonized calf, where the calf remained colonized for several weeks, shedding *C. difficile* in the environment. Two other calves, born on the farm (farm 14), and later throughout the study placed in the same individual box as the colonized calf, were found colonized with the same clonal *C. difficile* isolate. This is the first suggestion of a possible calf to calf and farm to farm transmission of *C. difficile*. However, due to lower sensitivity of the culture method compared to qPCR [36] and the history of calf trading between these two farms, we could also assume that transmission of this clonal *C. difficile* isolate could have happened before the start of our study.

Interestingly, one calf (T19 from farm 16) was positive for three consecutive times with *C. difficile* ribotype 033, which were not related (STRD≤10) or clonal (STRD ≤2). Calves have been shown to harbor different *C. difficile* ribotypes during different life stages [14]. Results of this study also indicate that they can be colonized with different MLVA types of the same *C. difficile* ribotype. Some environmental *C. difficile* strains (farm 5 and farms 2, 13, 14, 15) were clonal to strains from calves found on seemingly unrelated farms (farms 2, 4, 16 and farms 5, 6). There might be some epidemiological connection that we are unaware of, since farms have fields scattered across the area and sometimes cross paths with each other. Or simply, the high relatedness of all the samples tested could be the consequence of less natural variability in ribotype 033 than in other ribotypes as stated in the article from Bakker et al. [28]. Another possibility that could contribute to the spreading of different MLVA types of *C. difficile* in the community is flying insects. In our study, we found a fly on farm 5 to harbor the same clone of *C. difficile* as calves on farm 16 (distance between farms approximately 5 km).

## Conclusions

In conclusion, we demonstrated that calves can shed high loads of *C. difficile* from birth and that there is a non-linear statistically significant decrease of *C. difficile* prevalence and load with age. The superior prevalence of ribotype 033 compared to other ribotypes gained from a previous study [22] gave us the opportunity to assess the epidemiology of *C. difficile* between farms. We have suggested a farm to farm transmission through trading of a colonized calf. However, environmental and calves *C. difficile* strains from the same farm weren’t always related. Nevertheless, implementing better hygiene and management measures may help decrease the risk of spreading CA-CDI between animals and the community. Trading calves older than 3 weeks would decrease the possibility for *C. difficile* dissemination in the community not only because of lower prevalence, but also because of lower load of *C. difficile* in feces.

## Methods

### Material

Twenty mid-size family dairy farms with 9 to 40 cows located in Slovenian Prealps were included in this study. Farms run by family member only were selected based on several factors; location, accessibility, farmers compliance and number of dairy cows in production. All data with regards to the farms characteristics were described before [22]. Feces from all calves on the farm were collected at the time of sampling. All calves had mandatory ear tags used for individual identification. Fecal samples from calves (*n* = 2442) were collected individually from the rectum with clean latex gloves in two weeks intervals over a period of one year [22]. From these samples, 243 were positive for CD with qPCR and subsequently, 76 were identified as CD ribotype 033 with selective culture and ribotyping [22].

Environmental samples collected on every farm during each meteorological season were soil, manure, water, feed, other animals present on the farm, barn fly (*Stomoxys calcitrans*) and droppings from Barn Swallow (*Hirundo rustica*) [22]. Fourteen environment samples (manure = 7, soil = 6, barn fly (*Stomoxys calcitrans*) = 1) that were used in this study, were identified as CD ribotype 033 by selective culture and ribotyping [22].

### Methods

#### Quantification of *C. difficile* in calves fecal samples

Calves fecal samples were tested for *C. difficile* specific fragment of 16S gene using a quantitative real-time PCR (qPCR) reported by Bandelj et al. [36] with a LOD and LOQ of 7.72 CD cells/g feces and 77.2 CD cells/g feces, respectively. Samples (*n* = 243) were retested in duplicates and in 1:10 dilutions to evaluate for possible inhibitory effects of the matrix.

#### Multilocus variable-number tandem-repeat analysis (MLVA) of *C. difficile* ribotype 033

For MLVA, we used 90 *C. difficile* ribotype 033 isolates belonging to 76 calves and 14 environment samples. The *C. difficile* isolates were tested for relatedness with a modified MLVA. All MLVA PCRs for six loci were performed in singleplex format as described previously [27, 28]. To determine the genetic distance between isolates the minimum spanning tree was constructed. The number of differing loci and the summed tandem repeat difference (STRD) was used as coefficients for the genetic distance in BioNumerics, version 7.0 (Applied Maths) as previously described [37]. Genetically related were isolates with a STRD ≤10, whereas clonal complexes were defined by a STRD ≤2.

### Statistical analysis

The association between the prevalence and load of bacteria of *C. difficile* and age was estimated by the logistic regression model; the unit of analysis was an animal. Restricted cubic splines (using 5 knots) were used to account for a highly non-linear association between the prevalence/load of bacteria and age.

The association between the number of *C. difficile* and ribotype 033 positives and number of calves was estimated with Poisson generalized linear model with log link, including farm ID as a random effect (random intercept) and time of sampling as a fixed effect to account for repeated measurements; here the unit of analysis was a farm. For *C. difficile* a non-linear association was modeled by using restricted cubic splines with 3 knots. A possible non-linear association for ribotype 033 was also considered, however since the non-linear effect was not significant (*P* > 0.05), the results assuming a linear association are presented.

A *p*-value of less than 0.05 was considered as statistically significant. The analysis was performed with R language for statistical computing (R version 3.0.3) [38].

## Additional files

Additional file 1: Quantification results for *C. difficile* in feces of calves with single positive sample (0–21 days). (DOCX 35 kb)

Additional file 2: Quantification results for *C. difficile* in feces of calves with single positive sample (22–180 days). (DOCX 33 kb)

## Abbreviations

CA-CDI: Community acquired *Clostridioides (Clostridium) difficile* infection
CDI: *Clostridioides (Clostridium) difficile* infection
MLVA: Multilocus variable-number tandem-repeat analysis
qPCR: quantitative real-time PCR
STRD: Summed tandem repeat difference
