# Supplementary material for: Quantification of Clostridioides (Clostridium) difficile in feces of calves of different age and determination of predominant Clostridioides difficile ribotype 033 relatedness and transmission between family dairy farms using multilocus variable-number tandem-repeat analysis
Source: BMC Vet Res. 2018 Oct 1;14:298. doi: 10.1186/s12917-018-1616-8 (PMC6167908; doi:10.1186/s12917-018-1616-8)
Supplement: Supplementary file 1 — Quantification results for C. difficile in feces of calves with single positive sample (0–21 days). (DOCX 35 kb) [file 12917_2018_1616_MOESM1_ESM.docx]

Additional file 1: Quantification results for *C. difficile* in feces of calves with single positive sample (0-21 days)

| Age  (days) | No. *C. difficile* | | | | | | | | |
| --- | --- | --- | --- | --- | --- | --- | --- | --- | --- |
|  | 1 | 2 | 3 | 4 | 5 | 6 | 7 | 8 | 9 |
| 0 | 126 | 1063 | 324 | 2997 | 22058 |  |  |  |  |
| 1 | 453 | 2015 | 1010999 |  |  |  |  |  |  |
| 2 | 3673 | 3746 | 17778 | 26562 | 742 | LOQ |  |  |  |
| 3 | 14080 | 6854 | 631 | 148 | 15386 | 35678 | 1241893 | 793 |  |
| 4 | 14070 | 176509 | 3060471 | 1812 | 309534 | 206422 | 6279 | 465 | 387 |
| 5 | 769 | 3639 | 776376 | 6516 | 249096 | 618 |  |  |  |
| 6 | 966 | 24861 |  |  |  |  |  |  |  |
| 7 | 23203 | 3614 | 165202 |  |  |  |  |  |  |
| 8 | 896280 | 5440930 | 8504 | 1083 | 2303 | 562 | 9010042 | 1889085 | 667 |
| 9 | 2378 | 11188 | 932 | 1483 | LOQ |  |  |  |  |
| 10 | 1374886 | 45473 | 14966 | 1219 | 3425663 |  |  |  |  |
| 11 | 468 | 9341018 |  |  |  |  |  |  |  |
| 12 | 104 | 471 |  |  |  |  |  |  |  |
| 13 | 178 | 474 |  |  |  |  |  |  |  |
| 14 | 913 | 641 |  |  |  |  |  |  |  |
| 15 | 129 | 1399 |  |  |  |  |  |  |  |
| 16 | 657 | 28544 |  |  |  |  |  |  |  |
| 17 | 2707 | LOQ |  |  |  |  |  |  |  |
| 18 | / |  |  |  |  |  |  |  |  |
| 19 | 341 | 3188 |  |  |  |  |  |  |  |
| 20 | / |  |  |  |  |  |  |  |  |
| 21 | 21211 |  |  |  |  |  |  |  |  |

LOQ – under the limit of quantification
