# Supplementary material for: Quantification of Clostridioides (Clostridium) difficile in feces of calves of different age and determination of predominant Clostridioides difficile ribotype 033 relatedness and transmission between family dairy farms using multilocus variable-number tandem-repeat analysis
Source: BMC Vet Res. 2018 Oct 1;14:298. doi: 10.1186/s12917-018-1616-8 (PMC6167908; doi:10.1186/s12917-018-1616-8)
Supplement: Supplementary file 2 — Quantification results for C. difficile in feces of calves with single positive sample (22–180 days). (DOCX 33 kb) [file 12917_2018_1616_MOESM2_ESM.docx]

Additional file 2: Quantification results for *C. difficile* in feces of calves with single positive sample (22-180 days)

| Age (days) | No. *C. difficile* |
| --- | --- |
| 22 | 6254 |
| 23 | LOQ |
| 25 | LOQ |
| 26 | 22303 |
| 27 | 158 |
| 30 | LOQ |
| 33 | LOQ |
| 41 | 276 |
| 43 | 382 |
| 47 | LOQ |
| 48 | LOQ |
| 56 | LOQ |
| 67 | LOQ |
| 81 | LOQ |
| 82 | LOQ (2X) |
| 102 | LOQ |
| 107 | 579 |
| 114 | LOQ |
| 120 | LOQ |
| 127 | LOQ |
| 130 | 256 |
| 133 | 1677 |
| 136 | LOQ |
| 139 | 629 |
| 150 | 227 |
| 155 | 177 |
| 160 | 756 |
| 169 | LOQ |

LOQ- under the limit of quantification
